# Impact of La Concentration on Ferroelectricity of La-Doped HfO_2_ Epitaxial Thin Films

**DOI:** 10.1021/acsaelm.1c00672

**Published:** 2021-10-19

**Authors:** Tingfeng Song, Huan Tan, Romain Bachelet, Guillaume Saint-Girons, Ignasi Fina, Florencio Sánchez

**Affiliations:** † Institut de Ciència de Materials de Barcelona (ICMAB-CSIC), Campus UAB, Bellaterra, 08193 Barcelona, Spain; ‡ Univ. Lyon, Ecole Centrale de Lyon, INSA Lyon, CPE Lyon, CNRS, Institut des Nanotechnologies de Lyon - INL, UMR5270, Université Claude Bernard Lyon 1, 69134 Ecully, France

**Keywords:** ferroelectric HfO_2_, ferroelectric oxides, epitaxial HfO_2_, epitaxial oxides on silicon, thin films

## Abstract

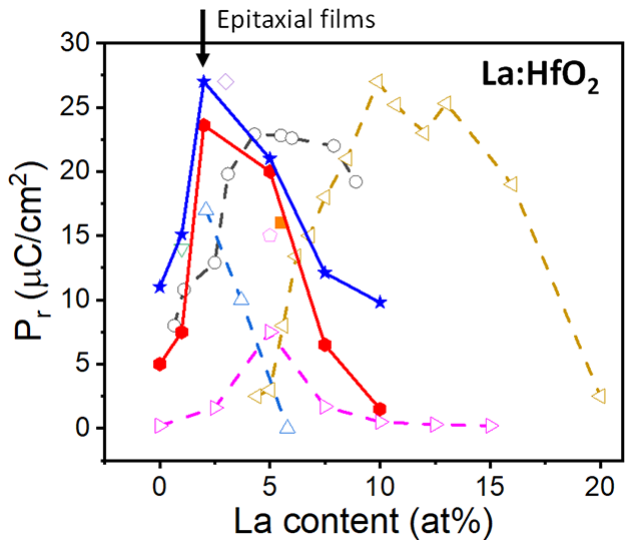

Epitaxial thin films of HfO_2_ doped with La have been grown on SrTiO_3_(001) and Si(001), and the impact of the La concentration on the stabilization of the ferroelectric phase has been determined. Films with 2–5 at. % La doping present the least amount of paraelectric monoclinic and cubic phases and exhibit the highest polarization, having a remanent polarization above 20 μC/cm^2^. The dopant concentration results in an important effect on the coercive field, which is reduced with increasing La content. Combined high polarization, high retention, and high endurance of at least 10^10^ cycles is obtained in 5 at. % La-doped films.

## 1 Introduction

Ten years after the first report on ferroelectricity in doped HfO_2_, this material is receiving a huge interest from the academy and industry. Most of the progress has been made by investigating polycrystalline films,^1−7^ and recently epitaxial films are also contributing.^8−15^ A significant number of chemical elements are used as dopant to stabilize the ferroelectric phase in polycrystalline HfO_2_. The most investigated chemical composition is Hf_0.5_Zr_0.5_O_2_.^16^ Recently, attention is being paid to large radius dopant atoms, particularly La, and high values of polarization and endurance have been reported.^5^ Moreover, robust ferroelectricity has been demonstrated in 1 μm thick La:HfO_2_ films.^17^

The dependence of the polarization of La:HfO_2_ films on the La content is reported in few papers, which are focused in polycrystalline films and with big difference in the determined optimal La content. Chernikova et al.^18^ grew by plasma-enhanced atomic layer deposition films of thickness *t* = 10 nm with La content ranging from 2.1 to 5.8 at. % and measured the highest remanent polarization (*P*_r_ = 17 μC/cm^2^) in the 2.1 atom % film. Schroeder et al.^19^ used atomic layer deposition (ALD) to grow *t* = 10 nm films with a wide range of La content from 4.4 to 34 at. % and reported the highest *P*_r_ (27 μC/cm^2^) for a content of around 10 at. %. More recently, Mart et al.^20^ prepared by ALD *t* = 10 nm films with La content from less than 1 to more than 8 at. %, measuring the largest *P*_r_ (23 μC/cm^2^) in films with a La concentration of around 5.5 at. %. TiN was used as bottom and top electrode in these studies. In contrast, Schenk et al.^17^ prepared *t* = 45 nm La:HfO_2_ films (La content ranging from 0 to 15 at. %) by chemical solution deposition using bottom and top Pt electrodes, and they reported the maximum *P*_r_ (7 μC/cm^2^) for films with a La content of 5 at. %. The dispersion in the reported results suggests that the optimal content of La depends on the thin film deposition technique and growth parameters, as noted by Materlik et al.^21^ Indeed, distinct deposition techniques and processing parameters result in different amount of oxygen vacancies, which has an impact on the crystallization of the competing polymorphs.^7^

In contrast to polycrystalline samples, epitaxial La:HfO_2_ films have been very scarcely investigated,^22,23^ and the effect of the La content on the ferroelectric properties has not been addressed. To investigate it, we have prepared a series of La:HfO_2_ films with varied La content (0,1, 2, 5, 7.5, and 10 at. %) deposited on (001)-oriented SrTiO_3_ (STO) and Si substrates. We present here characterization of the crystal phases and surface morphology and detailed electrical measurements that include polarization loops, leakage current, and permittivity loops. The impact of the La content on the crystal phases and the electrical properties is determined. The remanent polarization is the largest for La content of 2–5 at. %, and the coercive field decreases with La content. As a result of these dependences, a La concentration of 5 at. % allows a high endurance of more than 10^10^ cycles with good retention properties.

## 2 Experimental Section

**Figure 1:**
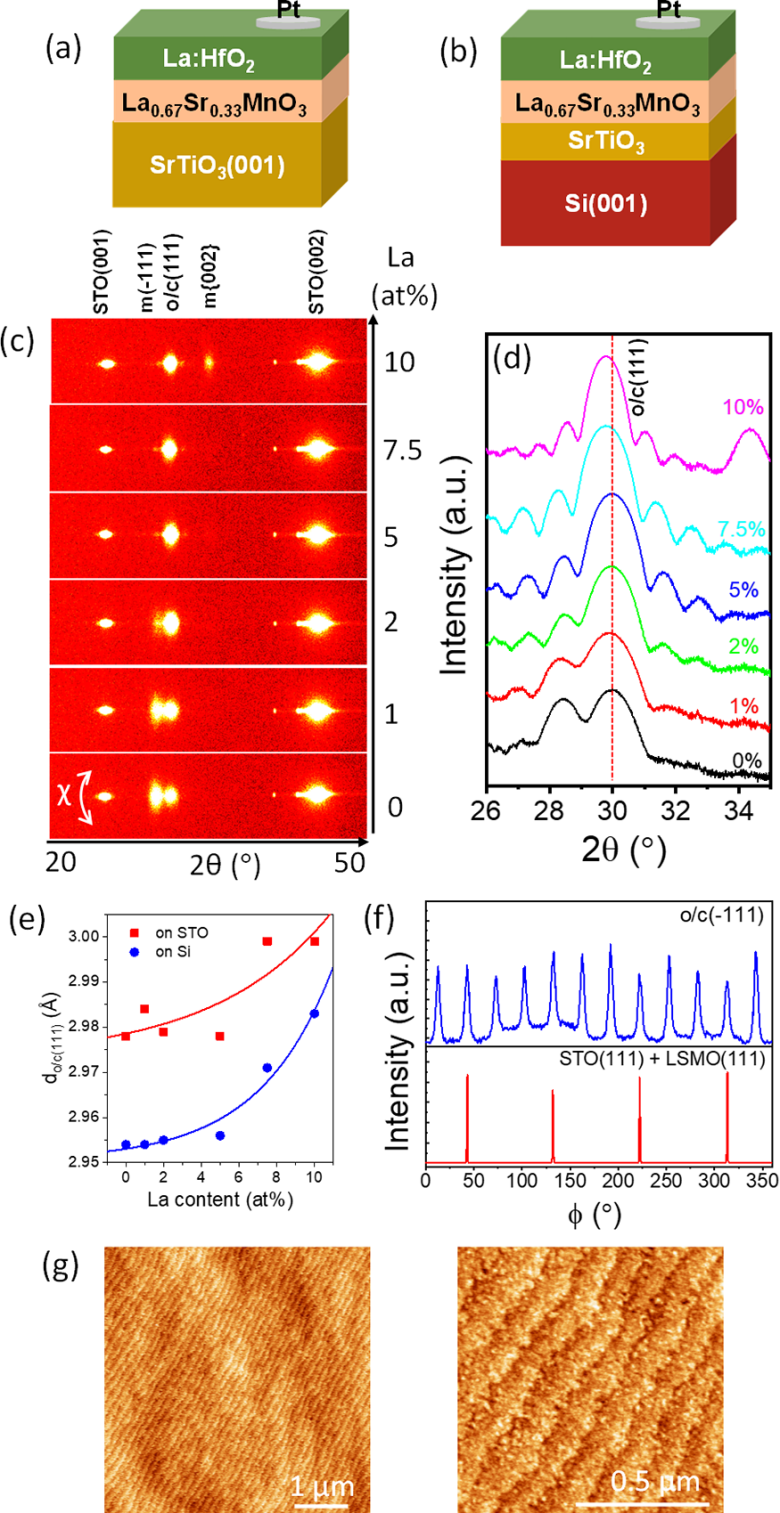
Sketches of the Pt/La:HfO_2_/LSMO heterostructures on STO(001) (a) and STO/Si(001) (b). XRD 2θ–χ maps (c) and θ–2θ scans (d) of the La:HfO_2_ films on STO(001). (e) Out-of-plane lattice parameter associated with the o/c(111) reflection as a function of the La concentration for films on STO(001) (red squares) and Si(001) (blue circles). Lines are guides for the eye. (f) XRD ϕ-scans, measured in the 2 at. % La film on STO(001), around o/c(−111) reflections (top panel) and STO(111)/LSMO(111) reflections. (g) Topographic AFM images of the 2 at. % La film on STO(001), scanned in 5 μm × 5 μm (left panel) and 1 μm × 1 μm (right panel) regions.

La:HfO_2_ films and bottom La_0.67_Sr_0.33_MnO_3_ (LSMO) electrodes were grown on STO(001) substrates in a single process by pulsed laser deposition (PLD) using a KrF excimer laser. Hf_1–*x*_La_*x*_O_2−δ_ (*x* = 0, 0.01, 0.02, 0.05, 0.075, and 0.1) and La_0.67_Sr_0.33_MnO_3_ sintered pellets of 1 in. diameter were used as targets. LSMO electrodes of thickness around 25 nm were grown at a substrate temperature *T*_s_ = 700 °C, an oxygen pressure *P*_O_2__ = 0.1 mbar, and a laser frequency of 5 Hz and have an electrical resistivity ∼1 mΩ·cm. The corresponding parameters used to grow La:HfO_2_ films of thickness around 8.5 nm were *T*_s_ = 800 °C, *P*_O_2__ = 0.1 mbar, and 2 Hz. An equivalent series of La:HfO_2_/LSMO bilayers were grown on Si(001) wafers (p-type, resistivity 1–10 Ω·cm) buffered with epitaxial STO films deposited *ex situ* by molecular beam epitaxy (MBE). Details of MBE deposition of the STO buffers are reported elsewhere.^24,25^ Platinum circular top electrodes, of thickness 20 nm and diameter 20 μm, were deposited *ex situ* by sputtering through stencil masks for electrical characterization. Figures 1a and 1b show sketches of the Pt/La:HfO_2_/LSMO heterostructures on STO(001) and STO/Si(001), respectively.

X-ray diffraction (XRD) with Cu Kα radiation was used to study the crystal structure. A Siemens D5000 and a Bruker D8-Discover equipped with a point detector and a Bruker D8-Advance equipped with a 2D detector diffractometer were used. A Keysight 5100 atomic force microscope (AFM) was used in dynamic mode to investigate the surface topography.

An AixACCT TFAnalyser2000 platform was used to measure ferroelectric polarization loops, current leakage, retention, and endurance at room temperature (except for some retention measurements performed at 85 °C). Measurements were done connecting the LSMO bottom electrode to the ground and biasing the top Pt contact. Ferroelectric polarization loops were obtained in dynamic leakage current compensation (DLCC) and positive-up negative-down (PUND) modes with a frequency of 1 kHz. Endurance was evaluated by using bipolar square pulses and measuring the loops by DLCC. Capacitance (*C*) loops were measured by using an impedance analyzer (HP4192LF, Agilent Co.) operated with an excitation voltage of 0.3 V at 50 kHz. Relative dielectric permittivity (ε_r_)–voltage loops were extracted from capacitance values by using the *C* = ε_0_ε_r_*A*/*t* relation, where *A* is the electrode area and *t* is the film thickness. Retention measurements were done by poling the sample (triangular pulse, 0.25 ms), measuring polarizations loops at 1 kHz by using the PUND protocol after a delay time, and determining the *P*_r_ from the first polarization curve (as described in the Supporting Information S1 and in ref (11)).

## 3 Results

XRD 2θ–χ maps (Figure 1c) show bright spots at χ = 0°. The spots at 2θ ∼23° and ∼47° correspond to (001) and (002) reflections of the STO substrate overlapped to the LSMO electrode reflection. The other spots correspond to La:HfO_2_ reflections. The spots at 2θ ∼28.3° and ∼34.4° are at the positions of the (−111) and {200} reflections, respectively, of the monoclinic (m) phase (space group number 14, *P*2_1_/*c*). The maps of all films show a spot at 2θ ∼ 30°, where the (111) reflection of the orthorhombic (o) phase (61, *Pbca*), the (111) reflection of the cubic (c) phase (225, *Fm*3*m*), and the (101) reflection of the tetragonal (t) phase (137, *P*4_2_/*nmc*) are located. In polycrystalline La:HfO_2_ films, the orthorhombic and cubic phases generally stabilize with moderately low and high La content, respectively.^18,19^ Here, for the sake of simplicity, the spot at 2θ ∼ 30° is indexed as o/c(111). The 2θ–χ map of the undoped HfO_2_ film (0 at. % La) shows a bright m(−111) spot while the m-{200} reflections are not detected. In films with a concentration of La 1 and 2 at. %, the intensity of the m(−111) spot decreases with increasing La content, and the m-{200} spot is barely visible. The m(−111) spot is not detected in films with higher La content, but a m-{200} spot is clearly observed in the 10 at. % La film. XRD θ–2θ scans measured with a point detector are shown in Figure 1d. There are Laue oscillations around the reflection at 2θ ∼ 30°. The films thickness and the out-of-plane lattice parameter corresponding to this reflection were determined from the simulation of the Laue oscillations (Supporting Information S2). The films are around 8 nm thick, except the 10 at. % film which is 10.5 nm thick. The *d*_o/c(111)_ values are ∼2.98 Å in the Hf_1–*x*_La_*x*_O_2−δ_ films with *x* up to 0.05 and slightly expanded, ∼3.0 Å, in the *x* = 0.075 and *x* = 0.1 films (Figure 1e). The *d*_o/c(111)_ elongation in La-rich films could signal an increased fraction of cubic phase in these films, as it has been observed in polycrystalline films.^18^ The XRD characterization of the equivalent series of films on Si(001) is summarized in Supporting Information S3. The θ–2θ scans show that the o/c(111) peak in all films is accompanied by a strong m(−111) peak in the pure HfO_2_ film and a low-intensity m{200} peak in the other samples. The *d*_o/c(111)_ out-of-plane lattice parameter shows a dependence on La content similar to that of films on STO(001) with lattice expansion in the La-rich films (Figure 1e). However, for a fixed La content, the *d*_o/c(111)_ parameter of the film on Si(001) is smaller than that of the corresponding film on STO(001). Similar shrinkage occurs in other doped-HfO_2_ epitaxial films on Si(001) and is considered an effect of the tensile stress caused by the Si(001) substrate. Si has a low thermal expansion coefficient, and this produces a tensile stress on the film when it is cooled after growth at high temperature.^26,27^

The ϕ-scans shown in Figure 1f confirm that the La:HfO_2_ films are epitaxial, presenting the same epitaxial relationship with the STO(001) substrate than other doped HfO_2_ epitaxial films grown on either perovskite or Si(001) substrates.^25,28,29^ Moreover, topographic AFM images (Figure 1g) confirm that the films are very flat, with morphology of terraces and steps and low root-mean-square roughness (rms) of around 1.5 Å. Topographic AFM images of the complete series of films with varying La concentration are shown in Supporting Information S4. All films are very flat, although terraces are barely visible in the 10 at. % film, and the rms roughness increases to 3.7 Å. The La:HfO_2_ films on STO/Si(001) are also very flat, with rms roughness around 2 Å (Supporting Information S5).

**Figure 2:**
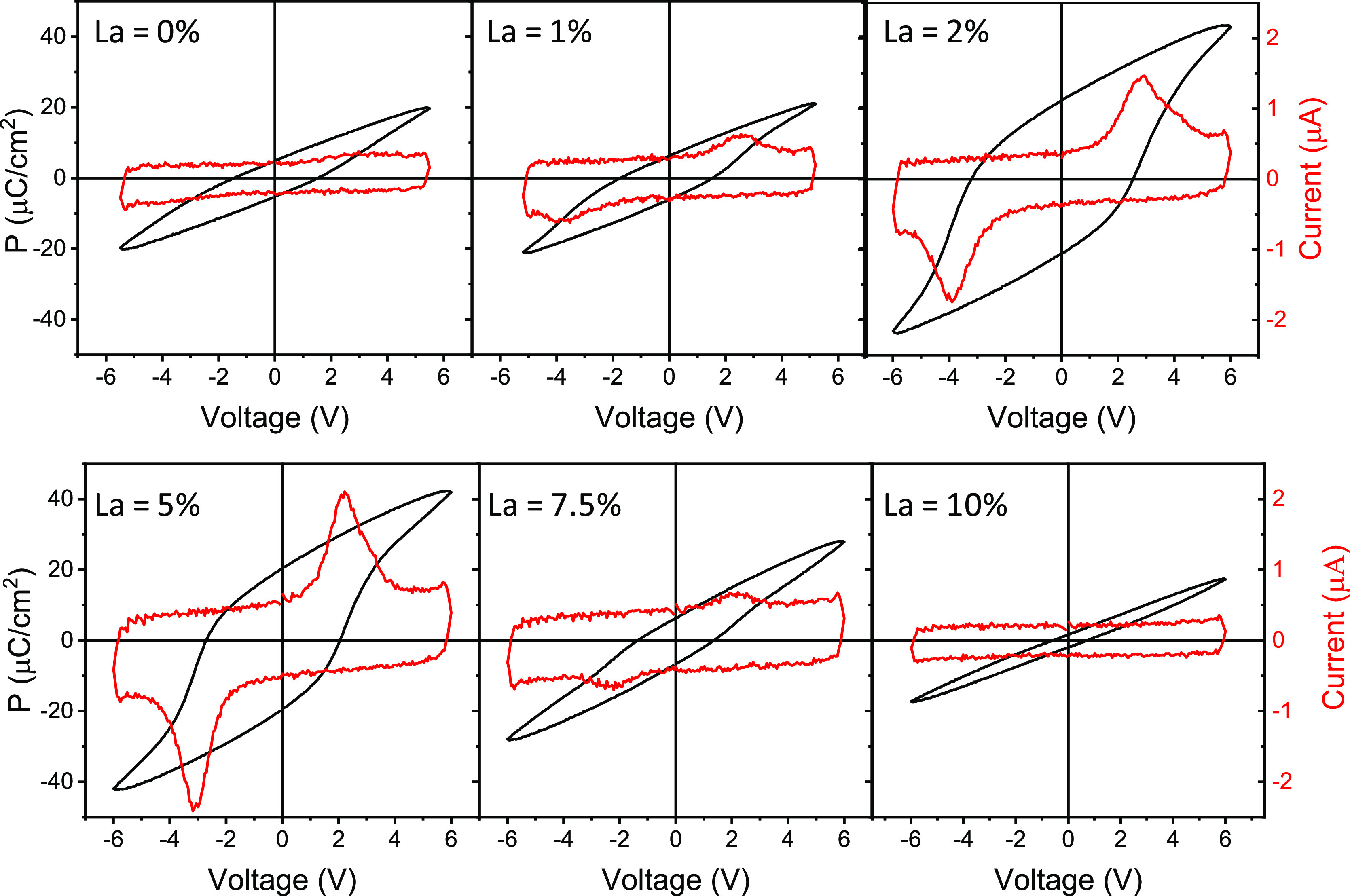
Current–voltage curves (red lines) and polarization loops (black lines) of the series of La:HfO_2_ films on STO(001). The La concentration is indicated in the label at the top left of each panel.

Figure 2 shows current–voltage curves (red lines) and polarization loops (black lines) of the films on STO(001), measured in the pristine state by the DLCC method. The switching peaks in the current–voltage curve of the pure HfO_2_ film are small, and remanent polarization (*P*_r_) is <5 μC/cm^2^. The amplitude of the ferroelectric switching peaks and the resulting polarization become greater with increasing La concentration, with *P*_r_ particularly high in the 2 at. % La (*P*_r_ ∼ 22 μC/cm^2^) and 5 at. % La (*P*_r_ ∼ 20 μC/cm^2^) films. The polarization decreases in the films with a higher amount of La, being *P*_r_ ∼ 6.5 μC/cm^2^ in the 7.5 at. % La film, while the 10 at. % La film does not show ferroelectric switching peaks. PUND measurements confirmed the enhancement of ferroelectric polarization in the films with La concentration 2–5 at. % (Supporting Information S6). The polarization of the films on Si(001) shows a similar dependence on the La concentration, with *P*_r_ above 25 and 20 μC/cm^2^ in the 2 and 5 at. % films, respectively (Supporting Information S7). Piezoelectric force microscopy characterization of the 2 and 5% doped samples (Supporting Information S8) also shows a ferroelectric response.

**Figure 3:**
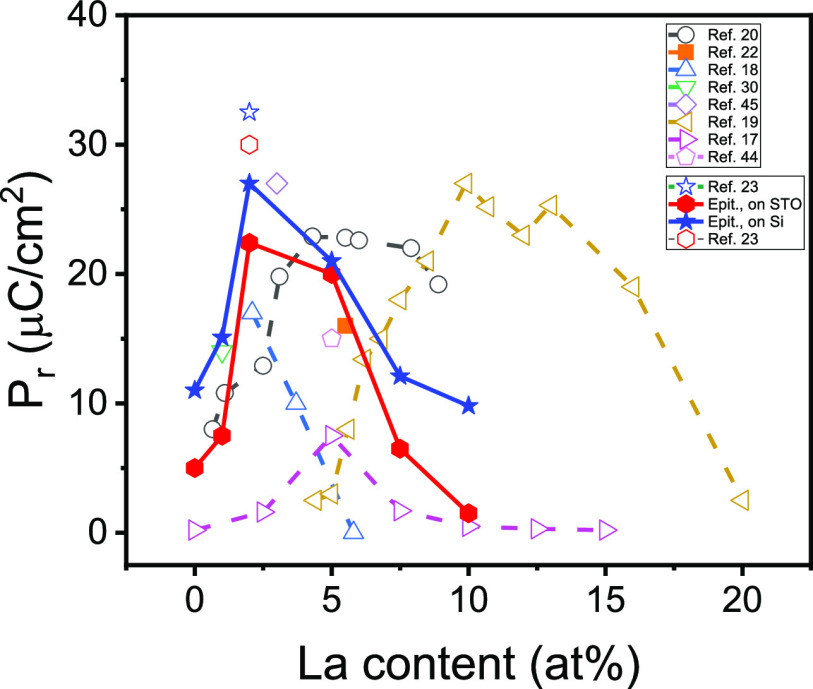
Dependence of the remanent polarization with the La concentration of the epitaxial films on STO(001) (solid red diamonds) and Si(001) (solid blue stars). The empty red diamond and empty blue star correspond to 2 at. % *t* = 6.9 nm epitaxial films grown by using same conditions on STO(001) and Si(001), respectively.^23^ The remanent polarization of a *t* = 12 nm epitaxial film on STO(001) reported by Li et al.^22^ is indicated by a solid orange square. Data of series of polycrystalline films with varied La concentration are shown with open gray circles (*t* = 10 nm),^20^ open up blue triangles (*t* = 10 nm),^18^ open pink right triangles (*t* = 45 nm),^17^ and open gold left triangles (*t* = 10 nm).^19^ Other reported data of polycrystalline films with fixed La concentration are indicated by open pink pentagon (*t* = 45 nm)^44^ and open violet diamond (*t* = 50 nm).^45^

Figure 3 shows the dependence of the remanent polarization of the epitaxial films on STO(001) (solid red rhombi) and Si(001) (solid blue diamonds) on the La concentration. The concentration of La that maximizes the polarization in the epitaxial films is seen to be in the range 2–5 at. %. The polarization of the 2 at. % epitaxial films is larger when the film thickness is <7 nm.^23^ On the other hand, the polarization reported by Li et al.^22^ for a *t* = 12 nm epitaxial 5.5 at. % film (solid orange square) fits well to our data. Figure 3 also includes data reported for series of polycrystalline films. The dependences on the La concentration for the polycrystalline films reported by Chernikova et al.^18^ (open blue triangles) and Schenk et al.^17^ (open pink triangles) do not differ greatly from the epitaxial films. In contrast, the data reported by Mart et al.^20^ (open gray circles) and particularly by Schroeder et al.^19^ (open gold triangles) point to a greater amount of La having the largest polarization. This can be due to the combined effect of several factors, including the possibility of La segregation depending on the preparation conditions. Because epitaxial films are closer to intrinsic HfO_2_ than polycrystalline films, a concentration of 2–5 at. % is estimated to be optimal to stabilize the ferroelectric phase in La-doped HfO_2_.

**Figure 4:**
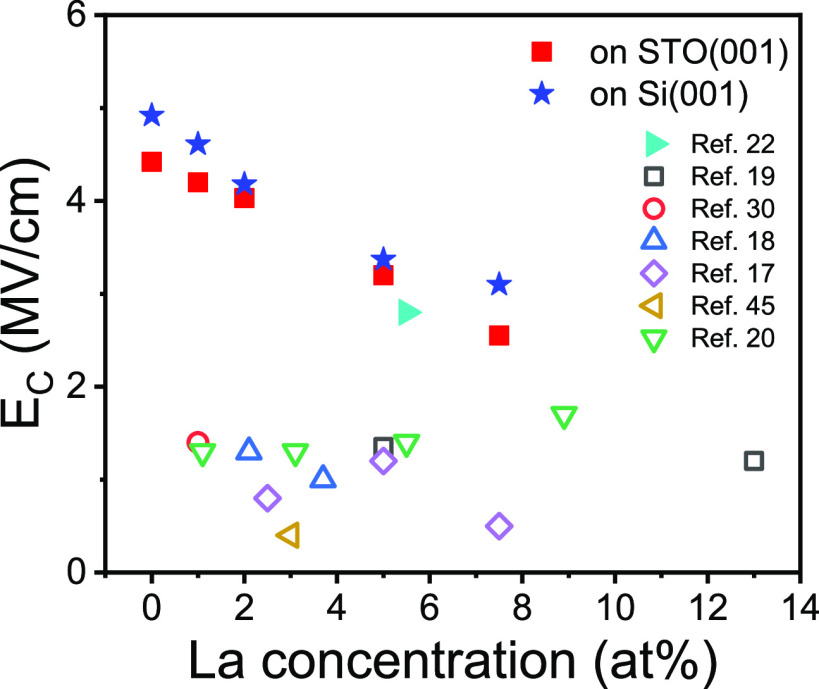
Coercive electric field dependence of La concentration for epitaxial films on STO(001) (solid red squares) and Si(001) (solid blue stars). Values were determined from the current peak position on the current–voltage curves measured by DLCC. The solid right green triangle indicates the *E*_C_ value reported for an epitaxial *t* = 12 nm film.^22^ Open symbols indicate reported *E*_C_ values for polycrystalline films: open black squares,^19^ open red circle,^30^ open up blue triangles,^18^ open purple diamonds,^17^ open left gold triangle,^45^ and open down green triangles.^20^ Values from ref 20 were taken from Figure 3b, and only the current peaks of greater amplitude in the films showing double peaks were considered.

The concentration of La has a great effect not only on polarization but also on the coercive field (*E*_C_). Figure 4 (round symbols) shows the *E*_C_ dependence of the films on STO(001) with the La concentration, determined from the peak position in current–voltage curves (Figure 2). The films have a larger coercive field than La-doped HfO_2_ polycrystalline films.^18−20,30^ This is in accordance with the usual high coercive field of epitaxial films of HfO_2_ doped with different atoms.^8^ The possibility of selecting *E*_C_ is of enormous interest, since it could allow to enhance the endurance. Thus, the monotonic decrease of *E*_C_ from 4.42 to 2.55 MV/cm with the concentration of La is remarkable. The same dependence is observed in the films on Si(001), with a decrease in *E*_C_ from 4.92 to 3.1 MV/cm (Figure 4, star symbols). The *E*_C_ value of an epitaxial La:HfO_2_ film on STO(001) reported by another group (solid right green triangle)^22^ fits very well with the *E*_C_–La content dependence of our epitaxial films. The decrease of the *E*_C_ of the epitaxial films with the increase in La content could be due to a greater number of defects that would facilitate domain switching.^19^ The *E*_C_ values of most polycrystalline films range from 0.5 to 1.7 MV/cm (Figure 4, empty symbols), without a clear dependence on La content. Schroeder et al.^19^ noted a slight decrease in *E*_C_ with La concentration (Figure 4, for simplicity, shows only two of the reported values). More recently, they have reported^31^*E*_C_ ∼ 3 MV/cm in *t* = 10 nm polycrystalline La:HfO_2_ films.

**Figure 5:**
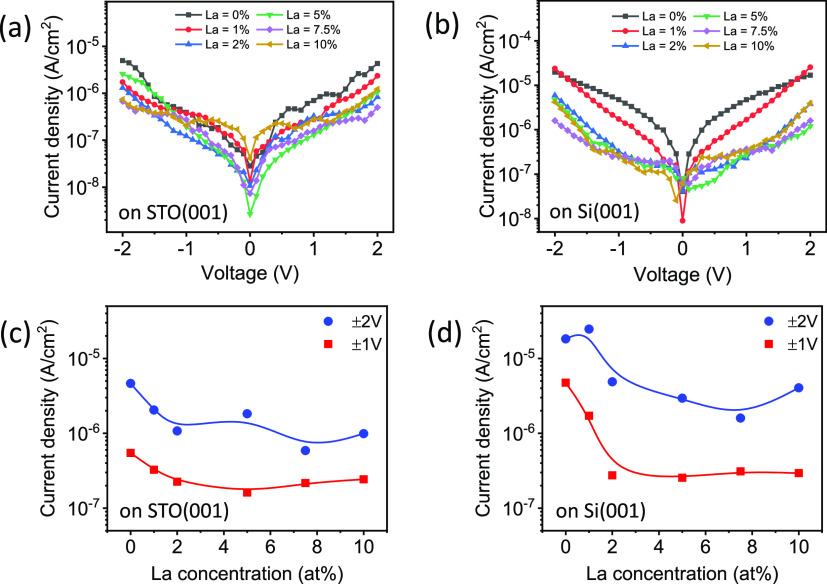
Current leakage curves of the films on STO(001) (a) and Si(001) (b). Dependence of the current leakage at 1 V (red squares) and 2 V (blue circles) on the La concentration in films on STO(001) (c) and Si(001) (d). The current leakage values in (b) and (c) are the average values at positive and negative bias.

Figure 4 shows a clear dependence of *E*_C_ with the concentration of La for epitaxial films and a great dispersion for the polycrystalline films. The defects present in polycrystalline films, expected in greater quantity than in epitaxial films, can be a main factor decreasing the *E*_C_ and causing dispersion.^32^ Polycrystalline films of HfO_2_ doped with different atoms tend to have a lower *E*_C_ compared to the equivalent epitaxial films.^6,8^ Furthermore, *E*_C_ ∼ *t*^–2/3^ scaling is reported for epitaxial films,^8,23,33^ but it has rarely been observed with polycrystalline films. An exception, recently reported by Materano et al.,^31^ shows a strong dependence of the coercive field of polycrystalline HZO and La:HfO_2_ (the concentration of La is not indicated) on thickness of the film and the grain size. The *E*_C_ values were very high, and the comparison with equivalent epitaxial HZO^33^ and La:HfO_2_^23^ (Supporting Information S9) evidences (1) the polycrystalline films (HZO and La:HfO_2_)^31^ exhibit *E*_C_ ∼ *t*^–2/3^ scaling and have *E*_C_ values similar to epitaxial films of the same thickness and (2) *E*_C_ values are slightly higher in La:HfO_2_ than in films of HZO of the same thickness. The leakage curves of the films on STO(001) and Si(001) are shown in Figures 5a and 5b, respectively. The pure HfO_2_ film (0 at. % La) on STO(001) exhibits leakage current of ∼5 × 10^–6^ and ∼5 × 10^–7^ A/cm^2^ at 2 and 1 V, respectively. In the doped films, leakage decreases down to ∼1 × 10^–6^ A/cm^2^ (at 2 V) and ∼2 × 10^–7^ A/cm^2^ (at 1 V) with La concentration >1 at. % (Figure 5c). The current leakage values are similar to those commonly reported in doped HfO_2_ polycrystalline films of similar thickness.^34^ However, they are high compared to the extremely low leakage current of ∼10^–8^ A/cm^2^ at 2 MV/cm that has been reported in polycrystalline films of TiN/La:HZO/TiN on Si(001).^35^ Epitaxial La:HfO_2_ films on Si(001) show a similar dependence (Figure 5d) than on STO(001), although the leakage current, particularly at high voltage, is greater than in the equivalent films on STO(001). Similarly larger leakage current in epitaxial doped HfO_2_ films on STO-buffered Si(001) was previously reported.^23,36^ Leakage is measured by applying rather long voltage pulses (around 1 s), and ionic current at grain boundaries may be a relevant contribution.^37,38^ The higher leakage of films on Si(001) could be due to differences in the grain boundaries density or in grain boundaries microstructure.

**Figure 6:**
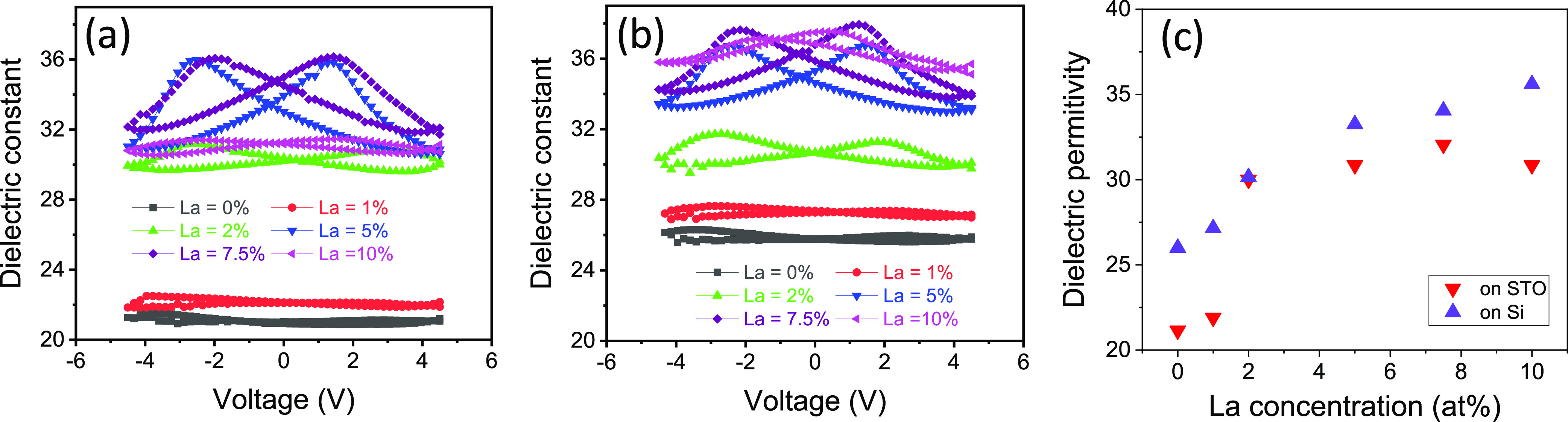
Dielectric constant loops of the films on STO(001) (a) and Si(001) (b). (c) Dependence of the dielectric constant at high field (average values at positive and negative bias) on the La concentration of films on STO(001) (red down triangles) and Si(001) (top blue triangles).

The dielectric polarization loops of the films on STO(001) and Si(001) are shown in Figures 6a and 6b, respectively. There is almost no hysteresis in undoped films and 1 at. % La films, and it is small in 10 at. % La films. In contrast, hysteresis is evident in films with La concentration in the 2–7.5 at. % range. The largest hysteresis is observed in 5 at. % La films, and 7.5 at. % films exhibit more pronounced hysteresis than films with 2 at. %. The permittivity loops of the samples with higher La concentration are more saturated due to lower coercive field. Therefore, hysteresis is more pronounced in films with lower La content showing higher ferroelectric polarization. There are also large differences in the dielectric constant values of the films. Figure 6c shows the dependence of the permittivity at high voltage as a function of La concentration for films on STO(001) (red down triangles) and Si(001) (blue up triangles). The dielectric constant is higher in the films on Si(001), and it increases with La concentration on both substrates, from 21–27 in undoped and 1 at. % films to 30–35 in films with La concentration higher than 5 at. %. The homogeneity of the samples was confirmed by measuring different capacitors of the 2 and 7.5 at. % La samples (Supporting Information S10). The dielectric constants of HfO_2_ polymorphs are reported to be around 24–29, 24–57, 36, and 19–25 for the orthorhombic, tetragonal, cubic, and monoclinic phases, respectively.^39,40^ The coexisting monoclinic phase (Figure 1a) in the undoped and lightly doped films explains the lower permittivity of these samples. Therefore, increasing La concentration results in the increase of the dielectric permittivity. Given that the cubic phase has the greatest dielectric permittivity, the dielectric permittivity increase with La content points to cubic phase formation in films with increasing La concentration, in agreement with observations on polycrystalline films.^18,19^ The crystalline phase evolution with the concentration of La, estimated from measurements of dielectric constant and XRD characterization, explains the dependence of *P*_r_ on the concentration of La (Figure 2). *P*_r_ is maximum in samples with 2–5 at. % La, being lower with lower and higher La content due to the greater amount of monoclinic and cubic paraelectric phases, respectively.

**Figure 7:**
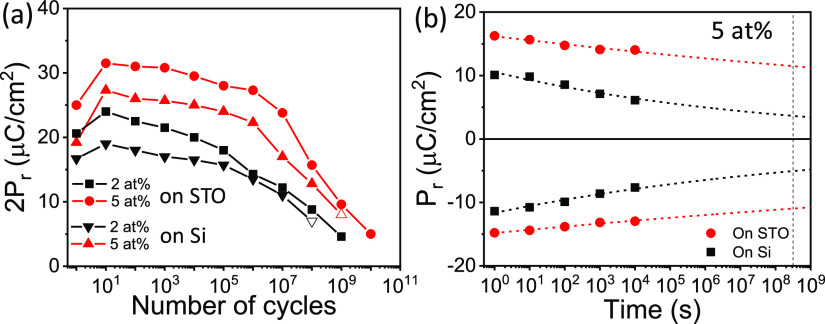
(a) Polarization window (2*P*_r_) as a function of the number of bipolar cycles of amplitude 5 V of films on STO(001) with La concentration 2 at. % (black squares) and 5 at. % (red circles) and 4.5 V of films on Si(001) with La concentration 2 at. % (black down triangles) and 5 at. % (red up triangles). (b) Retention of the 5 at. % film on STO(001) and Si(001) measured at 85 °C after being poled positively or negatively at 5 and 4.5 V, respectively. Red dashed lines are fits to *P*_r_ = *P*_0_*t*_d_^–*n*^ (see ref (23)). The vertical black dashed line marks a time of 10 years.

Figure 7a shows the endurance of the 2 and 5 at. % films on STO(001), which show the largest *P*_r_, measured in both cases by applying bipolar pulses of 5 V amplitude. Wake-up is limited in both cases to fewer than ten cycles. After these few initial cycles both films suffer fatigue but no dielectric breakdown. The 2*P*_r_ = 24 μC/cm^2^ of the La 2 at. % film after 10 cycles decreases to 14.3 μC/cm^2^ after 10^6^ cycles (41% polarization loss in 5 decades) and to 4.6 after 10^9^ cycles (19% of the value after 10 cycles). In contrast, the 5 at. % film presents higher 2*P*_r_ = 31.5 μC/cm^2^ after 10 cycles and decreases slowly to 27.3 μC/cm^2^ after 10^6^ cycles (13% polarization loss in 5 decades) and then more abruptly to 9.6 μC/cm^2^ after 10^9^ cycles and to 5 μC/cm^2^ after 10^10^ cycles. Note that the larger *P*_r_ and superior endurance at 5 V cycling voltage of the 5 at. % film are a consequence of the lower coercive field, which allows for more saturated ferroelectric switching with a field low enough to avoid dielectric breakdown and important fatigue. Figure 7a includes also the endurance test of the samples of the same composition grown on Si. It can be observed that sample breakdown occurs at 10^8^ and 10^9^ (empty symbols) for 2% and 5% doping, respectively. The occurrence of sample breakdown is probably related to the higher leakage of samples grown on Si compared to those grown on STO. Interestingly, the endurance of the here reported epitaxial film is comparable to the best reported in the polycrystalline ones,^18^ in spite of the larger cycling voltage used here. This indicates that the lower defects amount in epitaxial films is crucial to allow electric cycling in harder conditions than in polycrystalline ones. Besides large endurance, applications require high retention of capacitors poled by using same electric field that in endurance measurements and for both polarities. The 5 at. % film, in addition of endurance above 10^10^ cycles, exhibits excellent retention at 85 °C (see retention measurements methodology in the Supporting Information S1), with extrapolated polarization beyond 10 years for both up and down polarization states after being poled at 5 V (Figure 7b). The retention measurement performed on the 5% sample grown on Si is also shown in Figure 7b, and similar good retention is observed at expenses of the mentioned *P*_r_ reduction. The same state (SS), new same state (NSS), and opposite state (OS) retention measurements at room temperature of the 5% sample grown on STO^41^ using different writing times have been performed. These are shown in Supporting Information S11. It can be observed that retention does depend on writing time pulse, indicating the presence of fluid imprint field.^42,43^

## 4 Conclusions

In summary, epitaxial films of La-doped HfO_2_ have been grown on LSMO buffered STO(001) and Si(001), and the impact of the La concentration on crystalline phases and electrical properties has been determined. There is large amount of monoclinic phase in lightly doped films, while the cubic phase appears in high La content films. All films show very smooth surfaces, confirming the potential usefulness of epitaxial films in devices based in ultrathin layers or in complex heterostructures. Films with 2–5 at. % La are mostly orthorhombic and have a high remanent polarization above 20 μC/cm^2^. The coercive field of the films on both substrates decreases with the concentration of La. The 5 at. % film on STO(001), which combines high polarization and a lower coercive field, has an endurance of 10^10^ cycles and negligible wake-up effect, with high extrapolated polarization after 10 years at 85 °C. The equivalent film on Si(001) has long retention also and slight wake-up effect, but leakage current is higher and the endurance is limited to 10^9^ cycles.

## Supporting Information Available

Retention measurements methodology; simulation of Laue oscillations; XRD θ–2θ scans of the films on Si(001); topographic AFM images of films on STO(001); topographic AFM images of films on Si(001); PUND measurement of the films on STO(001); PUND measurement of the films on Si(001); piezoelectric force microscopy characterization; *E*_C_ dependence on thickness in polycrystalline and epitaxial films; dielectric loops measured in different capacitors; retention measurement with different pulse widths (PDF)
