# Supplementary material for: Impact of La Concentration on Ferroelectricity of La-Doped HfO2 Epitaxial Thin Films
Source: ACS Appl Electron Mater. 2021 Oct 19;3(11):4809–16. doi: 10.1021/acsaelm.1c00672 (PMC8613842; doi:10.1021/acsaelm.1c00672)
Supplement: Supplementary file 1 — el1c00672_si_001.pdf [file el1c00672_si_001.pdf]

Supporting Information

Impact of La concentration on ferroelectricity of La-doped HfO_2 epitaxial thin films

Tingfeng Song,[1] Huan Tan,[1] Romain Bachelet,[2] Guillaume Saint-Girons,[2] Ignasi Fina,[1,*] and Florencio Sánchez,[1,*]

[1] Institut de Ciència de Materials de Barcelona (ICMAB-CSIC), Campus UAB, Bellaterra 08193, Barcelona, Spain.

[2] Univ. Lyon, Ecole Centrale de Lyon, INSA Lyon, Université Claude Bernard Lyon 1, CPE Lyon, CNRS, Institut des Nanotechnologies de Lyon - INL, UMR5270, 69134 Ecully, France.

* ifina@icmab.es, fsanchez@icmab.es

Supporting information S1: Retention measurements methodology.

Figure S1 shows the pulse sequence for retention experiments shown in Figure 7(b). A prepoling pulse is applied to a single junction in the sample. This sets the initial polarization state. After delay time a complete PUND sequence is applied. The remanent polarization after the indicated delay time is obtained from the subtraction of the integrated polarization measured during U (containing the non-switchable contributions) to that measured during P (containing the non-switchable and switchable contributions). Thus, the remanent polarization only contains switchable contribution. For opposite polarization direction retention experiments the polarity of all the pulses of the sequence is inverted.

.

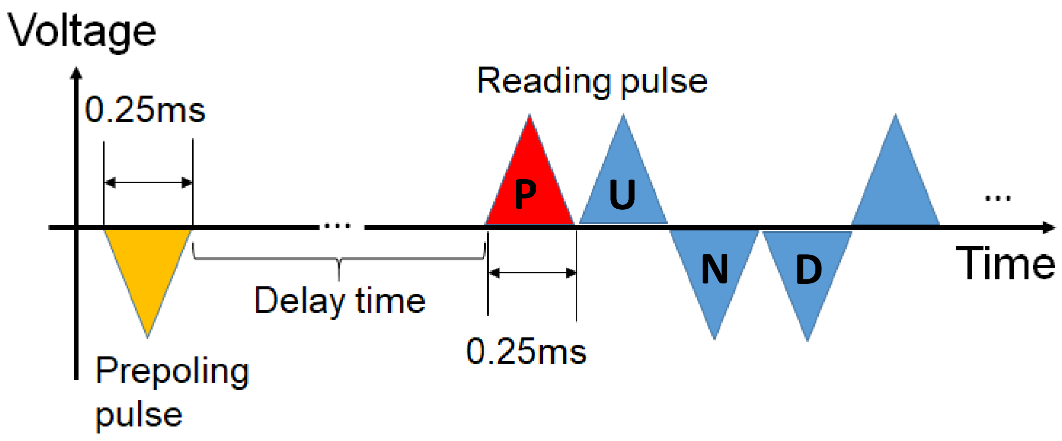

Figure S1. Pulse sequence used to perform retention experiments shown in the main text.

Supporting information S2: Simulation of Laue oscillations.

Figure S2 shows the XRD θ-2θ scans of the films on STO(001), with the simulation (red curves) of the o/c-(111) reflection according to the Laue equation

$$I(Q) = \left(\frac{\sin\left(\frac{QNc}{2}\right)}{\sin\left(\frac{Qc}{2}\right)} \right)^2$$

where Q = 4πsin(θ)/λ is the reciprocal space vector, N the number of unit cells along the out-of-plane direction and c the corresponding lattice parameter. The thickness of the films is around 8 nm, except the 10 at% La doped film that is about 10 nm. For example, the parameters to simulate the 5 at% doped film are N=28 and c=2.978 Å, corresponding to a thickness of about 8.3 nm.

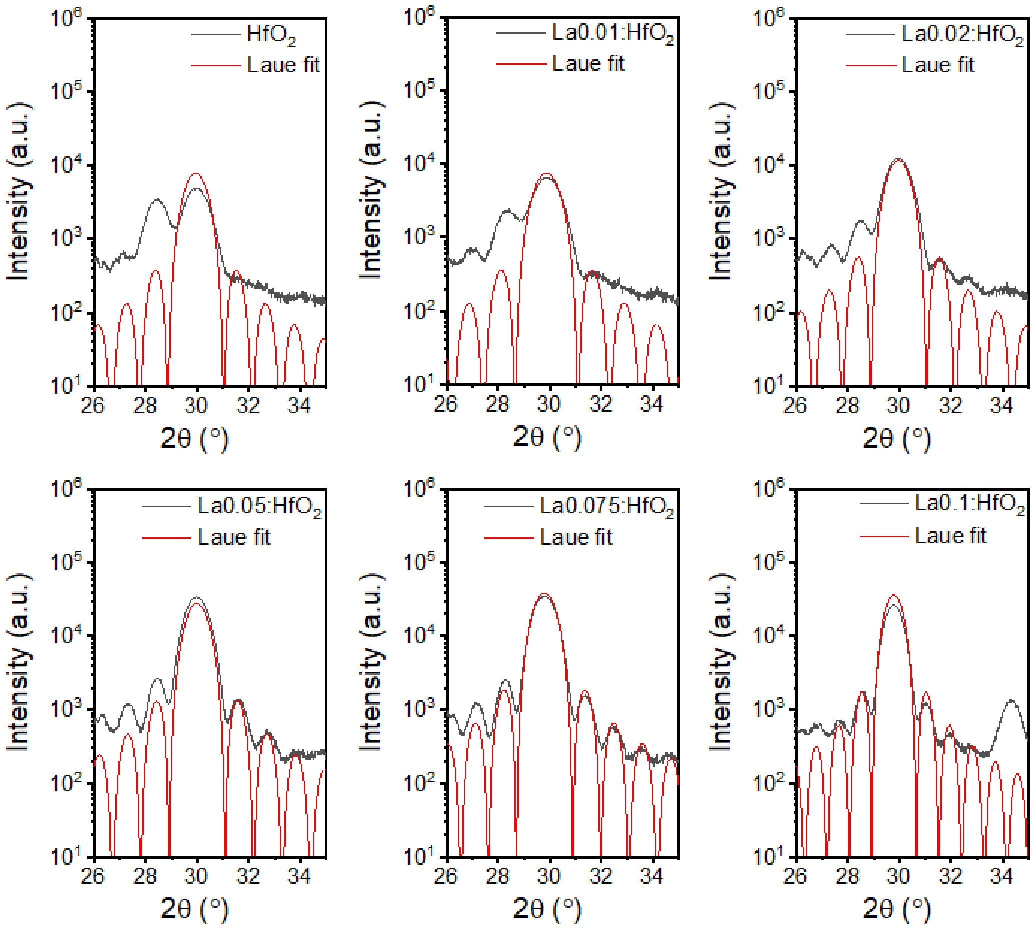

Figure S2. XRD θ-2θ scans of films with different La concentration on STO(001). Red curves are simulations of Laue oscillations.

Supporting information S3: XRD θ-2θ scans of the films on Si(001).

Figure S3 shows XRD θ-2θ scans of the films on Si(001). It can be observed that there is a shift of the o/c(111) peak towards smaller 2θ value with the increase of the La content. This could be due to the formation of cubic phase in the films with higher La content. The thickness of the films, estimated by Laue simulations, is around 8 nm.

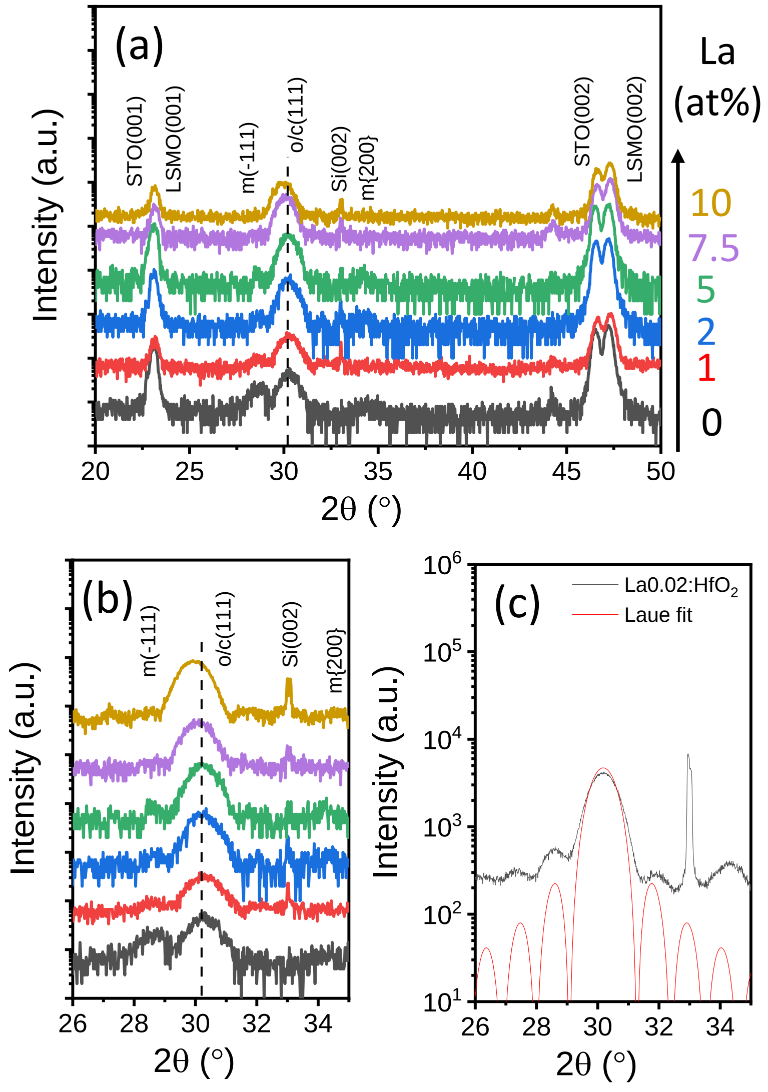

Figure S3. (a) XRD θ-2θ scans of the films on Si(001). (b) Enlarged range around o/c(111) peak. (c) Laue simulation of 2 at% La doped film.

Supporting information S4: Topographic AFM images of films on STO(001).

Figure S4 shows topographic AFM images of the films on LSMO/STO(001). The films are very flat, and terrace and steps can be observed in films with La content from 1 to 7.5 at%, with a rms roughness less than 2 Å. The 10 at% doped film is slightly rougher with rsm=3.7 Å.

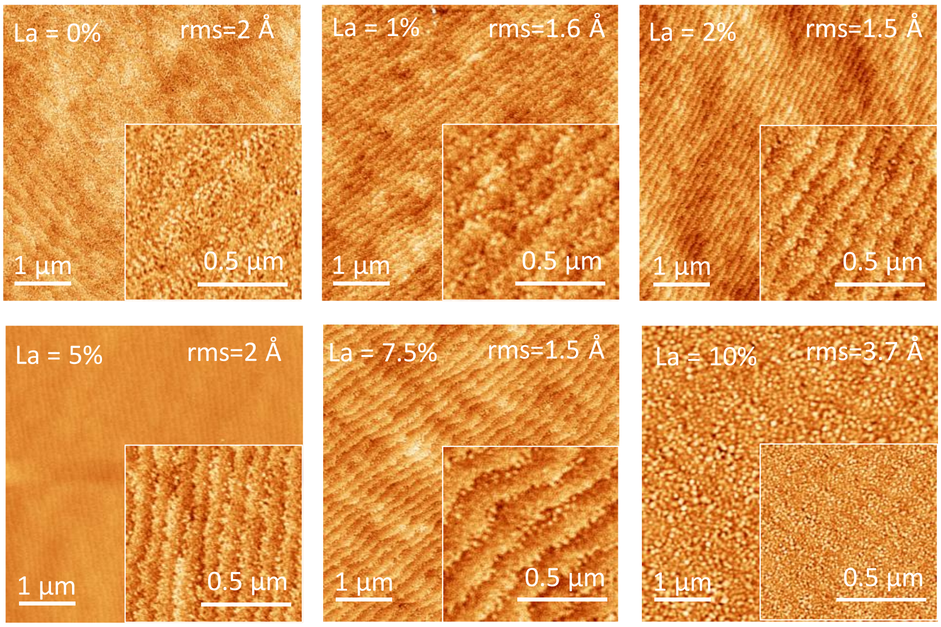

Figure S4. Topographic AFM images, 5 µm × 5 µm, of the films on STO(001). The inset in each image is a 1 µm × 1 µm scanned area.

Supporting information S5: Topographic AFM images of films on Si(001).

Figure S5 shows topographic AFM images and height profiles of the films on LSMO/STO/Si(001). The films are very flat, with a rms roughness less than 0.21 nm.

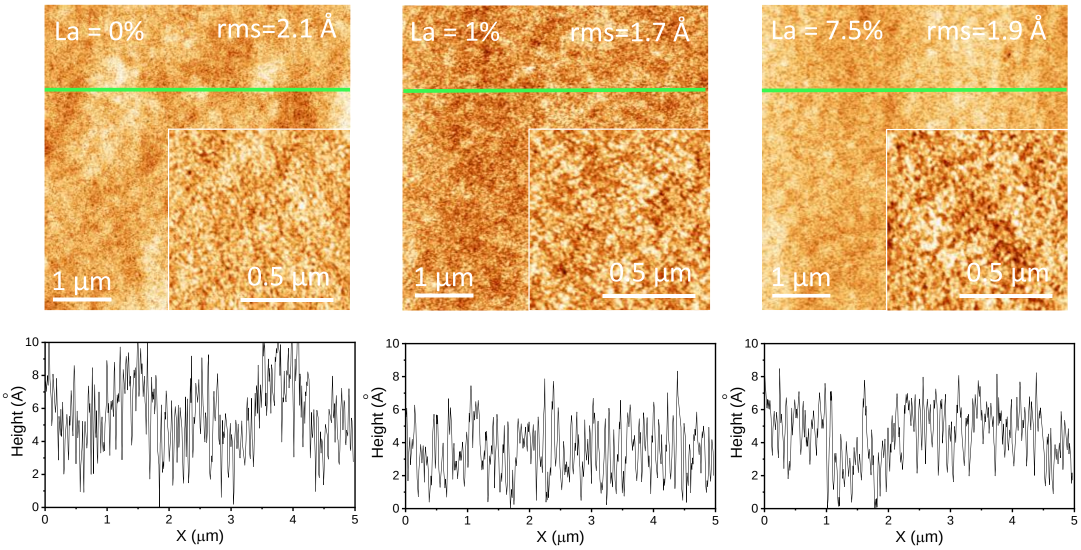

Figure S5. Topographic AFM images, 5 µm × 5 µm, of the films on LSMO/STO/Si(001). A height profile along the marked horizontal green lines is shown below each image. The inset in each image is a 1 µm × 1 µm scanned area.

Supporting information S6: PUND measurement of the films on STO(001).

Figure S6 shows polarization loops of the films on STO(001), measured by the PUND method. The remanent polarization is slightly smaller than that measured by the DLCC method. The optimal La concentration is 2 - 5 at%, presenting these films remanent polarization higher than 20 µC/cm^2.

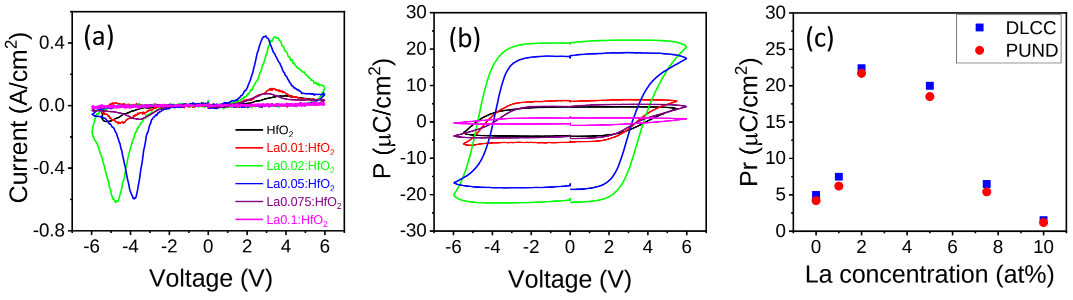

Figure S6. Polarization loops measured by PUND method, (a) current-voltage curves, (b) polarization-voltage curves, and (c) remanent polarization measured by PUND and DLCC methods.

Supporting information S7: PUND measurement of the films on Si(001).

Figure S7 shows polarization loops of the films on Si(001), measured by PUND and DLCC methods. The 2 at% and 5 at% La doped films have the highest remanent polarization, above 20 μC/cm^2.

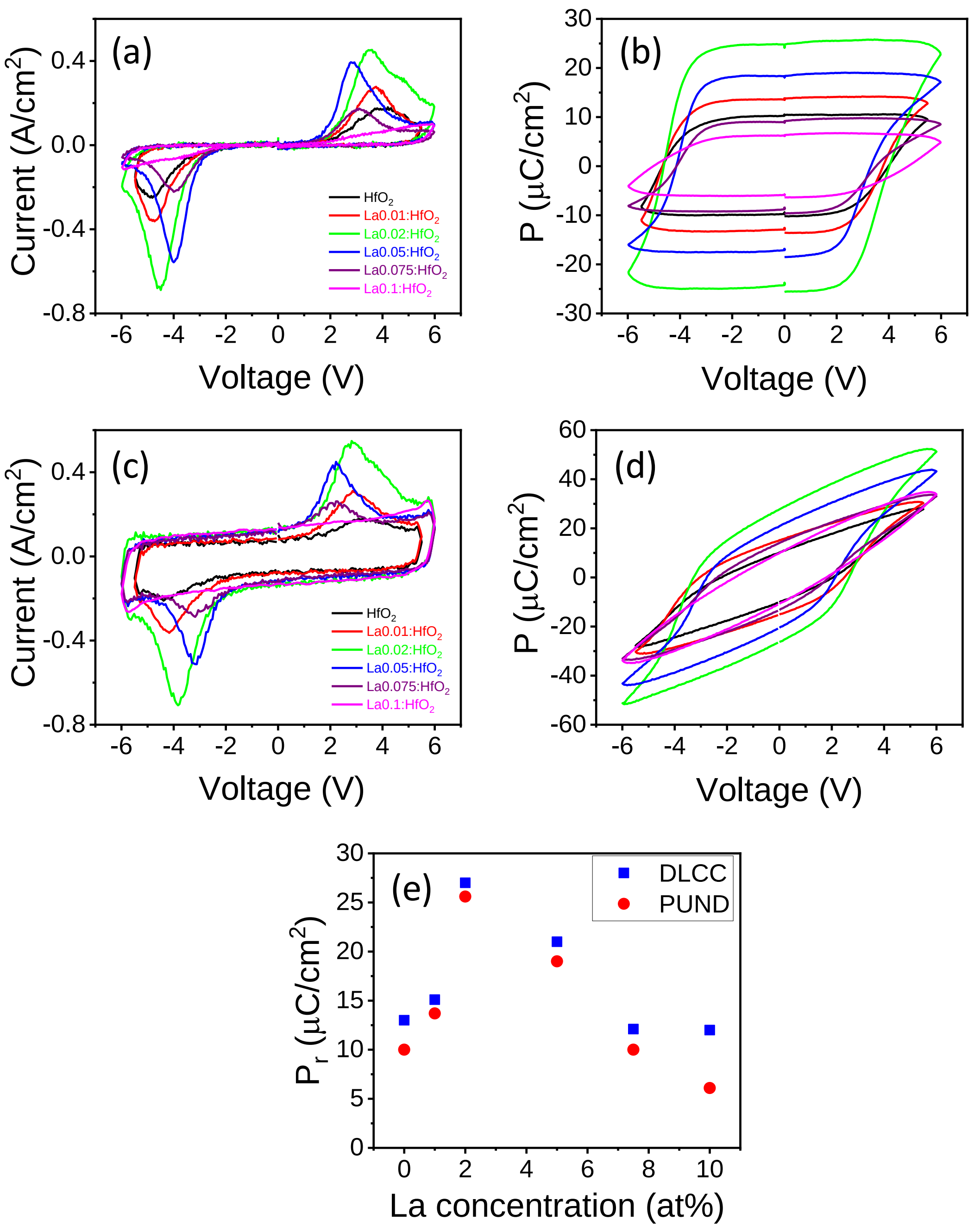

Figure S7. Current-voltage curves (a) and polarization-voltage loops (b) of the films on Si(001) measured by PUND. Current-voltage curves and polarization loops measured by DLCC are in (c) and (d), respectively. (e) Remanent polarization measured by PUND and DLCC as a function of the La concentration.

Supporting information S8: Piezoelectric force microscopy characterization.

Piezo-response force microscopy measurements of 2 at% and 5 at% La doped films were performed with an MFP-3D Asylum Research microscope (Oxford Instrument Co.), using the BudgetSensors silicon (n-type) cantilevers with Pt both sides coating (Multi75E-G). To achieve better sensitivity, the dual AC resonance tracking (DART) method was employed. Due to the difficulties quantifying PFM response in DART, piezoelectric coefficient has not been evaluated although the measurements in the two samples are done in the same conditions making them comparable. In Figure S8a,b, amplitude and phase for the 5 at% doped sample are show, respectively, after prepoling the sample with the microscope tip with ±8V in the indicated regions. In Figure S7a, the piezo-response amplitude is constant irrespectively of the prepoling voltage. In Figure S8b, it can be observed that piezo response phase contrast is 180°. In addition, the phase in the non pre-poled region is similar to that observed after prepoling with +8 V, thus indicating that the polarization in the as-grown state points towards LSMO in agreement with the small imprint observed in P-E loops. In Figure S8c,d, amplitude and phase piezo-response for the 2 at% doped are shown. Similar results to those obtained for the 5 at% sample are obtained. The small differences of the amplitude response between samples is below the variability observed among different measurements, i.e. not significative.

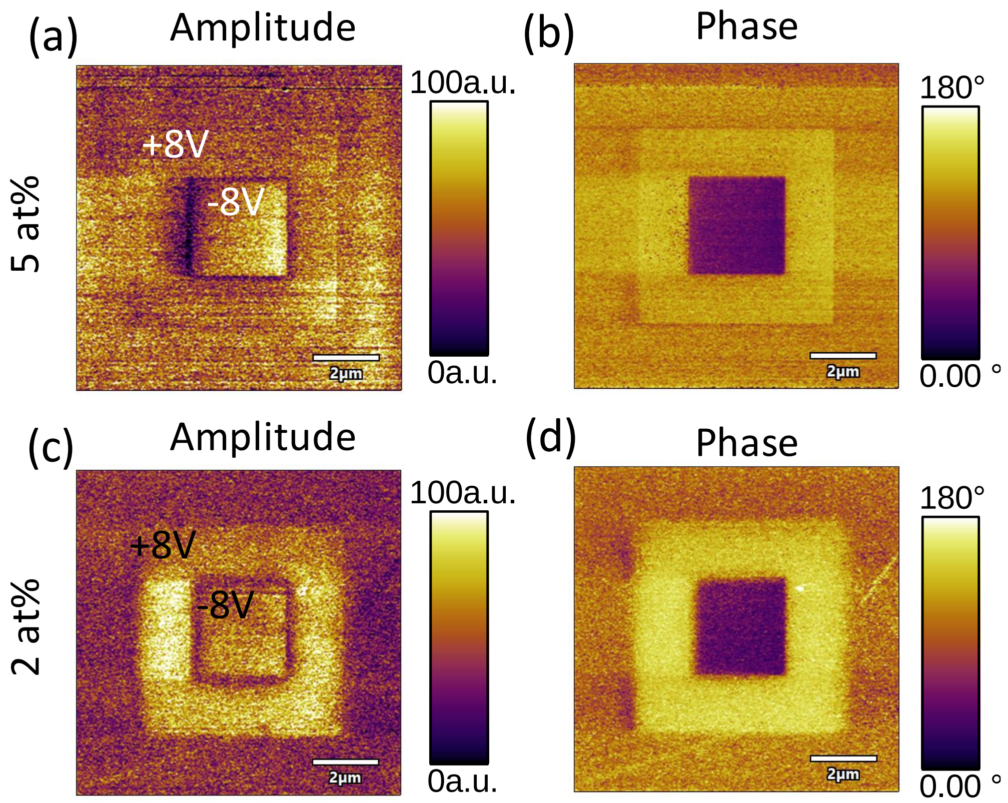

Figure S8. (a,b) Amplitude, phase images, respectively, collected on the 5 at% La doped HfO_2 film. (c,d) Amplitude, phase images, respectively, collected on the 2 at% doped HfO_2 film.

Supporting information S9: E_C dependence on thickness in polycrystalline and epitaxial films

E_C values on recently reported polycrystalline films[1] are very high, and similar to those of equivalent epitaxial films of same thickness.[2,3] Figure S9 shows the E_C values reported by Materano et al.[1] for polycrystalline films and we include the values of epitaxial HZO[4] and La:HfO_2[5] films. A very similar trend can be observed.

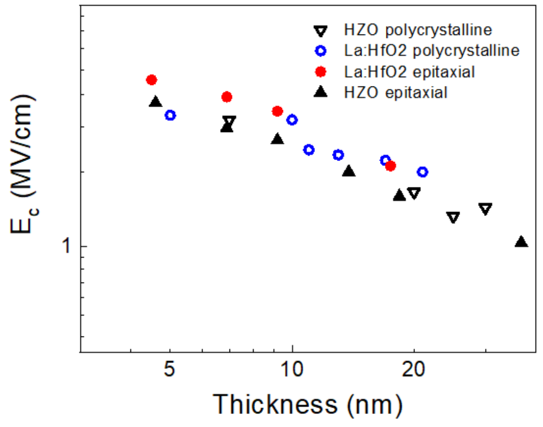

Figure S9. Comparison of E_C values that we report for epitaxial HZO films[4] and $La:HfO_2$ films[5] films and the ones reported for polycrystalline HZO and $La:HfO_2$ films by Materano et al.[1]

Supporting information S10: Dielectric loops measured in different capacitors.

Measurement of dielectric loops in different capacitors of the 2 at% and 7.5 at% films on STO(001) confirmed the homogeneity of the samples.

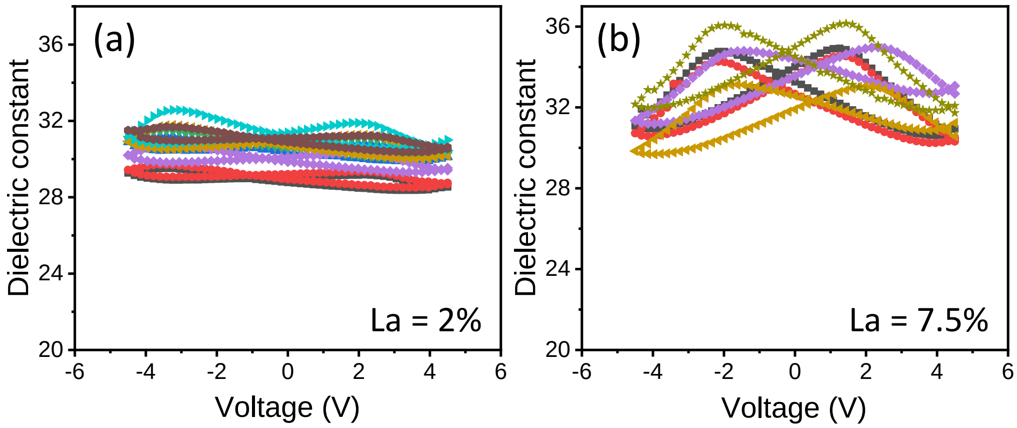

Figure S10. Dielectric loops of (a) 2 at% and (b) 7.5 at% films on STO, measured in different capacitors.

Supporting information S11: Retention measurement with different pulse width.

The same state (SS), new same state (NSS), and opposite state (OS) retention measurements of the 5 at% sample are shown in Figure S10a,b for different pulse width. In Figure S11a, it can be observed that SS and NSS the retention is extremely good for the explored delay time. The OS, as seen in polycrystalline films, shows poorer retention. Figure S11b shows that the retention is poorer if shorter pulses are used for the OS. For the SS and NSS the retention is similar to that observed if longer pulses are used.

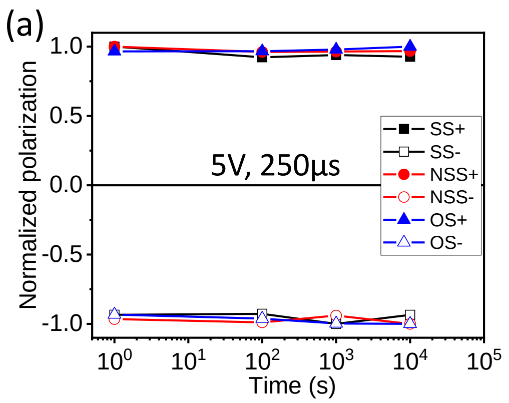

Figure S11. SS, NSS and OS retention measurements performed at room temperature using (a) 250 μs and (b) 50 μs writing pulses for the 5 at% sample.

References

(1) Materano, M.; Lomenzo, P. D.; Mulaosmanovic, H.; Hoffmann, M.; Toriumi, A.; Mikolajick, T.; Schroeder, U. Polarization Switching in Thin Doped HfO_2 Ferroelectric Layers. *Appl. Phys. Lett.* **2020**, *117*, 262904.

(2) Park, M. H.; Lee, D. H.; Yang, K.; Park, J. Y.; Yu, G. T.; Park, H. W.; Materano, M.; Mittmann, T.; Lomenzo, P. D.; Mikolajick, T.; Schroeder, U.; Hwang, C. S. Review of Defect Chemistry in Fluorite-Structure Ferroelectrics for Future Electronic Devices. *J. Mater. Chem. C* **2020**, *8* (31), 10526–10550.

(3) Fina, I.; Sánchez, F. Epitaxial Ferroelectric HfO_2 Films: Growth, Properties, and Devices. *ACS Appl. Electron. Mater.* **2021**, *3* (4), 1530–1549.

(4) Lyu, J.; Fina, I.; Solanas, R.; Fontcuberta, J.; Sánchez, F. Growth Window of Ferroelectric Epitaxial $Hf_{0.5}Zr_{0.5}O_2$ Thin Films. *ACS Appl. Electron. Mater.* **2019**, *1* (2), 220–228.

(5) Song, T.; Bachelet, R.; Saint-Girons, G.; Dix, N.; Fina, I.; Sanchez, F. Thickness Effect on Ferroelectric Properties of La-Doped HfO_2 Epitaxial Films down to 4.5 nm. *J. Mater. Chem. C* **2021**, *1* (207890), 3777.
